# Mr Sluggish Schizophrenia

**DOI:** 10.1192/bjb.2021.66

**Published:** 2021-08

**Authors:** Robert van Voren

**Affiliations:** Chief Executive, Federation Global Initiative on Psychiatry, The Netherlands. Email: rvvoren@gip-global.org

**Figure:**
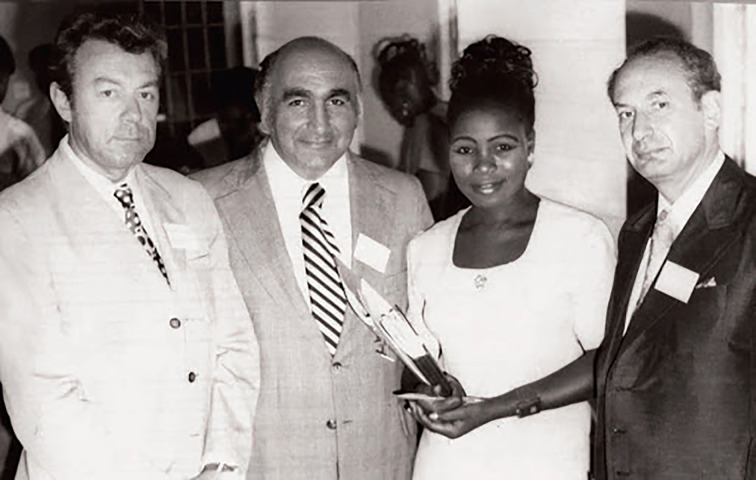
r.t.l. Anatoly Smulevich, unknown, Marat Vartanyan, Aleksandre Tiganov. Photo Credit: Robert Van Voren

On 15 April 2021, the World Psychiatric Association (WPA) congratulated the Russian professor Anatoly Smulevich on his 90th birthday on its website. It wrote that ‘his work is highly regarded both in Russia and around the world … the WPA would like to wish Prof. A.B. Smulevich the happiest of birthdays and thank him for his long-standing contribution to the world of psychiatry’.

To most, the name Smulevich rings no bells. I have been involved in the fight against the political abuse of psychiatry for almost 45 years and know full well that he was one of the main researchers in the field of ‘sluggish schizophrenia’, the main diagnosis used against dissidents in the Soviet Union. He belonged to the small group of ‘nomenklatura psychiatrists’ who monopolised all contacts with the outside world and dominated psychiatry in the USSR. Their actions caused the forced hospitalisation of thousands of dissenters, who were subjected to torturous ‘treatment’, and established a repressive and abusive psychiatry that affected millions of people and disabled many for the rest of their lives. Like the Nazi euthanasia programme, this Soviet political abuse of psychiatry is among the worst violations of medical ethics. Professor Smulevich not only contributed significantly to the ‘scientific basis’ of this perversion of medicine, he also actively defended the position that criticism against Soviet psychiatry was a matter of politically inspired slanderous allegations. He was considered by the KGB to be a ‘trusted psychiatrist’.

In an interview on 29 September 1986, he claimed that in the USSR ‘a sane person cannot be sent to a psychiatric hospital’. In March 1989, during a meeting with an American delegation, he explained that 38.1% of all schizophrenics suffered from sluggish schizophrenia and described the symptoms, which included ‘anti-Soviet thinking’ and ‘delusions of reformism’. He then argued that all dissidents could be diagnosed as suffering from sluggish schizophrenia. During the discussion of the case of an alleged victim, Smulevich continued: ‘In the Soviet system this is a person who has overvalued ideas. Yes, one can fight for freedom in a thousand and one ways but it should not be to the neglect of other areas of his life. [He] fought for freedom and even wrote books about freedom, neglecting all other ideas in his life. His life became unbalanced, all of his interests were expressed in one area’. In the same year, he published an article in the *British Journal of Psychiatry*, actively defending the concept of sluggish schizophrenia.

In most of the biographies of Professor Smulevich that one can now find on the internet, most of his work on sluggish schizophrenia and his activities during the Soviet period are omitted, but those who were sent to psychiatric hospitals because of their convictions very well remember the man nicknamed ‘Mr Sluggish Schizophrenia’.

